# Comparison of short- and long-term outcomes of bariatric surgery methods: A retrospective study

**DOI:** 10.1097/MD.0000000000030679

**Published:** 2022-09-23

**Authors:** Hasan Cantay, Kenan Binnetoglu, Umut Eren Erdogdu, Yurdakul Deniz Firat, Haci Murat Cayci

**Affiliations:** a Department of General Surgery, Kafkas University Faculty of Medicine, Kars, Turkey; b Department of General Surgery, The University of Health Sciences, Bursa Yuksek Ihtisas Research and Training Hospital, Bursa, Turkey.

**Keywords:** bariatric surgery, obesity, one anastomosis gastric bypass, Roux-En-Y gastric bypass, sleeve gastrectomy

## Abstract

The present study is intended to retrospectively compare the short- and long-term outcomes of 3 different treatment methods in patients undergoing bariatric surgery and the variances in weight and nutritional parameters during the preoperative and postoperative periods. In this study, 534 patients who underwent laparoscopic sleeve gastrectomy (LSG), laparoscopic Roux-En-Y gastric bypass (LRYGB), and laparoscopic one anastomosis gastric bypass (LOAGB) between 2014 and 2021 were included. The sociodemographic and biodemographic characteristics of these patients, their weight losses and nutritional changes in the preoperative and postoperative periods, operative times, hospital stays, complications, and morbidity and mortality rates were retrospectively compared. There was a statistically significant difference between the surgical methods in the percentages of excess weight loss and total weight loss in the 1st and 3rd months. There were significant differences in the homeostasis model assessment of insulin resistance, folic acid, vitamin D, iron, ferritin, and parathyroid hormone levels (*P* < .05). All 3 techniques were found to be successful in facilitating weight loss at the end of the first year. LRYGB and LOAGB were found to be superior to LSG in terms of remission from diabetes during the first 6 months, whereas LSG was superior to the other methods in terms of nutritional deficiencies. Despite being more advantageous in terms of operative time, LSG and LOAGB were disadvantageous compared with the LRYGB technique because of the higher rates of leakage and mortality in the LSG technique and the higher rate of bile reflux in the LOAGB technique.

## 1. Introduction

Obesity is defined as abnormal or excessive fat accumulation in the body to the extent that it impairs health and it remains a public health threat that is rapidly spreading globally. Obesity is known to be a risk factor for many diseases, such as coronary artery disease, hypertension, hyperlipidemia, cancer, and stroke, especially for insulin resistance. Therefore, obesity treatment bears significant importance as it is a serious health problem.^[1,2]^ Alternative options, such as diet, physical exercise, behavioral therapy, medical treatment, and surgery, have been explored to manage obesity. Effective weight loss with non-surgical treatment is attainable only in 3%–9% of the patients.^[3,4]^

In surgical treatment, “restrictive surgery” (such as sleeve gastrectomy and gastric banding) and “malabsorbative surgery” (such as jejunoileal bypass and duodenal switch) can be used either individually or together well (Roux-en-Y gastric bypass, biliopancreatic diversion, etc.).^[4–6]^ For years, numerous surgical methods have been compared in terms of their advantages and disadvantages; however, a gold-standard treatment option has not yet been developed.^[4–7]^

To establish whether any of the 3 different treatment methods studied herein, that is, laparoscopic sleeve gastrectomy (LSG), laparoscopic Roux-En-Y gastric bypass (LRYGB), and LOAGB, have any superiorities over each other, the present study retrospectively compared these methods in terms of their short- and long-term outcomes and variances in weight loss and nutritional parameters during the preoperative and postoperative periods.

## 2. Materials and Methods

The research data were obtained retrospectively from the records of the patients operated for morbid obesity at the General Surgery Clinics of Kafkas University Faculty of Medicine, The University of Health Sciences Bursa Training and Research Hospital. A total of 551 patients who underwent LSG, LRYGB, and LOAGB operations for morbid obesity between 2014 and 2021 were included.

Operation criteria: The operation criteria were BMI (body mass index) ≥35 kg/m^2^ patients with presence of 2 or more comorbidities, and BMI ≥40 kg/m^2^ patients with or without the presence of comorbidity.

The patient characteristics of age, sex, presence of comorbidity, history of operations, weight, BMI, smoking, employment status, American Society of Anesthesiologists (ASA) score, surgical technique, operative time, hospital stay, presence of complications, treatment for the complications, morbidity, and mortality were obtained from the records.

Along with these characteristics, the patient records were reviewed for data such as height, weight, BMI, fasting blood glucose (FBG), fasting insulin level, C-peptide, HbA1C, hemoglobin, triglycerides (TG), low density lipoprotein (LDL), high density lipoprotein (HDL), vitamin B12, folate, albumin, vitamin D, parathyroid hormone (PTH), Fe, and ferritin levels in the preoperative months 1, 3, 6 and 12. A total of 534 patients whose data were accessed were included in the current study. Seventeen patients with poor data quality were excluded from the study.

### 2.1. Assessment of weight loss

Weight loss assessment was performed using the percentages of excess weight loss (EWL) and weight loss (TWL). EWL% was calculated as follows: [(initial weight − postoperative weight)/(initial weight − ideal body weight)] × 100. The ideal body weight was defined as the weight corresponding to a BMI of 25 kg/m^2^. TWL% was calculated as follows: [(initial weight − postoperative weight)/(initial weight)]×100. The Clavien–Dindo complication classification system was used to evaluate the postoperative complications.^[8]^ The homeostasis model assessment of insulin resistance (HOMA-IR) score was calculated at each follow-up visit.^[9]^ HOMA-IR was calculated as follows: (Glucose × Insulin)/405.

*Ethics Committee Approval:* The necessary study approval was obtained from the Ethics Committee of Kafkas University Faculty of Medicine (approval no. 80576354-050-99/195, dated 22.09.2021)

### 2.2. Analysis of the research data

Data analysis was performed using the software SPSS version 20 for Windows package program. In the analyses, frequency and percentage values were utilized as the descriptive criteria and mean values as the criteria indicating location. The normality tests for the continuous variables were performed using the Shapiro–Wilk test. The normally-distributed variables were tested using analysis of variance (ANOVA), whereas the non-normally-distributed variables were tested using Kruskal–Wallis analysis of variance. Tukey test for the ANOVA and Tamhane T2 test for the Kruskal–Wallis analysis of variance were employed as post hoc analyses. Chi-squared test was performed to analyze the categorical data. *P* < .05 was considered statistically significant.

## 3. Results

### 3.1. Preoperative evaluation

According to the data shown in Table 1, the surgical methods demonstrated statistically significant differences in terms of sex, history of operation and ASA score (*P* < .05), whereas there are no statistically significant differences in terms of smoking, employment status, age, weight, and BMI (*P* > .05). While LSG had the highest rate of preference in both groups of men and women, LSG had a higher rate of preference in the group of women (64.1%) than in the group of men (45.9%). The LSG method showed a higher rate of preference (53.5%) in those with a history of operation. Additionally, when assessed based on the ASA score, the LSG method was preferred at higher rates in the groups of patients with ASA1 and ASA2 scores (80.4% and 66.4%). On the contrary, the LRYGB (37.4%) and LOAGB (38.5%) techniques had higher rates of preference in patients with ASA3 score (Table 1).

**Table 1:** Distribution of sociodemographic and biodemographic characteristics on surgical methods.

		LSG (n = 329) (%)	LRYGB (n = 109) (%)	LMGB (n = 96) (%)	*P*
Gender	Male	34 (45.9)	24 (32.4)	16 (21.6)	**.006**
	Female	295 (64.1)	85 (18.5)	80 (17.4)	
Smoking	Yes	99 (58.9)	42 (25.0)	27 (16.1)	.191
	No	230 (62.8)	67 (18.3)	69 (18.9)	
Employment status	Yes	189 (61.6)	59 (19.2)	59 (19.2)	.567
	No	137 (61.2)	50 (22.3)	37 (16.5)	
History of operation*	Yes	146 (53.5)	71 (26.0)	56 (20.5)	**.001**
	No	183 (70.1)	38 (14.6)	40 (15.3)	
ASA score†	ASA 1	74 (80.4)	11 (12.0)	7 (7.6)	**.001**
	ASA 2	233 (66.4)	64 (18.2)	54 (15.4)	
	ASA 3	22 (24.2)	23 (37.4)	35 (38.5)	
Age	Mean ± S.D.	41.0 ± 9.5	41.6 ± 9.8	42.7 ± 10.9	.414
Weight (kg)	Mean ± S.D.	123.2 ± 17.8	123.1 ± 19.6	119.8 ± 16.4	.206
BMI (kg/m^2^)	Mean ± S.D.	46.0 ± 5.6	44.8 ± 5.6	45.2 ± 5.5	.066

Significant differences (*P* < .05).

BMI = body mass index, LRYGB = laparoscopic Roux-En-Y gastric bypass, LSG = laparoscopic sleeve gastrectomy, S.D. = standard deviation.

* 3 data missing.

† ASA (American Society of Anesthesiologists) score.

### 3.2. Comorbidities

When assessed based on the effects of comorbidities on the surgical method preferences, the surgical methods were found to have statistically significant differences in terms of hyperlipidemia, hypertension, diabetes mellitus (DM), and sleep apnea syndrome (*P* < .05) (Table 2).

**Table 2:** Distribution of comorbidities on surgical methods.

		LSG	LRYGB	LMGB	Total	*P*
		N (%)*	N (%)*	N (%)*	N (%)†	
Hyperlipidemia	Yes	148 (55.8)	65 (24.5)	52 (19.6)	265 (49.6)	**.018**
	No	181 (67.3)	44 (16.4)	44 (16.4)	269 (50.4)	
Hypertension	Yes	70 (52.6)	27 (20.3)	36 (27.1)	133 (24.9)	**.005**
	No	259 (64.6)	82 (20.4)	60 (15.0)	401 (75.1)	
Diabetes mellitus (DM)	Yes	52 (37.4)	43 (30.9)	44 (31.7)	139 (26.0)	**.001**
	No	277 (70.1)	66 (16.7)	52 (13.2)	395 (74.0)	
Respiratory diseases	Yes	24 (80.0)	3 (10.0)	3 (10.0)	30 (5.6)	.102
	No	305 (60.5)	106 (21.0)	93 (18.5)	504 (94.4)	
Sleep apnea syndrome	Yes	11 (36.7)	13 (43.3)	6 (20.0)	30 (5.6)	**.003**
	No	318 (63.1)	96 (19.0)	90 (17.9)	504 (94.4)	
Heart diseases	Yes	7 (43.8)	4 (25.0)	5 (31.3)	16 (3.0)	.267
	No	322 (62.2)	105 (20.3)	91 (17.6)	518 (97.0)	
Thyroid diseases	Yes	12 (75.0)	4 (25.0)	0 (0.0)	16 (3.0)	.164
	No	317 (61.2)	105 (20.3)	96 (18.5)	518 (97.0)	
Collagen tissue disease	Yes	5 (50.0)	3 (30.0)	2 (20.0)	10 (1.9)	.703
	No	324 (61.8)	106 (20.2)	94 (17.9)	524 (98.1)	
PCOS	Yes	2 (50.0)	2 (50.0)	0 (0.0)	4 (0.7)	.281
	No	327 (61.7)	107 (20.2)	96 (18.1)	530 (99.3)	
kidney diseases	Yes	4 (100.0)	0 (0.0)	0 (0.0)	4 (0.7)	.285
	No	325 (61.3)	109 (20.6)	96 (18.1)	530 (99.3)	
Total					534 (100.0)	

Significant differences (*P* < .05).

PCOS = polycystic ovary syndrome, LRYGB = laparoscopic Roux-En-Y gastric bypass, LSG = laparoscopic sleeve gastrectomy.

* Row percentage.

† Column percentage

### 3.3. Weight loss

The treatment methods did not differ at a statistically significant level in terms of initial weight (*P* = .226). The EWL% and TWL% values of the 1st and 3rd months were found to present statistically significant differences between the surgical methods (*P* < .05). There were, however, no significant differences in the 6th and 12th months (*P* > .05). The post hoc analysis revealed that the mean values of EWL% and TWL% in the 1st and 3rd months were higher in patients who underwent LOAGB operation than in those who underwent LSG and LRYGB operations. However, the mean values of EWL% and TWL% in the 6th and 12th months were found to be similar in all 3 groups (Table 3).

**Table 3:** Comparison of weight loss according to surgical techniques.

	LSG	LRYGB	LMGB	*P*
**Initial weight (kg**)	123.2 ± 17.8	123.1 ± 19.6	119.8 ± 16.4	.226
**% EWL**				
1st mo	23.7 ± 11.9	21.6 ± 9.5	28.0 ± 11.7	**.001**
3rd mo	46.0 ± 14.8	43.4 ± 15.1	51.7 ± 14.8	**.001**
6th mo	65.7 ± 17.5	65.4 ± 19.1	70.4 ± 15.4	.090
1 yr	79.0 ± 19.4	79.3 ± 23.8	84.2 ± 17.2	.151
**% TWL**				
1st mo	10.5 ± 5.2	9.4 ± 4.0	12.1 ± 5.4	**.001**
3rd mo	20.4 ± 5.7	18.9 ± 5.3	22.3 ± 6.2	**.008**
6th mo	29.1 ± 6.4	27.8 ± 7.2	30.4 ± 5.7	.133
1 yr	35.2 ± 7.7	33.7 ± 9.0	36.5 ± 6.8	.179

Data are expressed as mean ± S.D. Significant differences (*P* < .05).

% EWL = the percentage of excess weight loss, % TWL = the percentage of total weight loss, EWL = excess weight loss, LRYGB = laparoscopic Roux-En-Y gastric bypass, LSG = laparoscopic sleeve gastrectomy.

### 3.4. Glycemic variables

The surgical methods did not exhibit any significant differences in the FBG, C-peptide, or HbA1c levels measured in the preoperative period and the postoperative months 1, 3, 6, and 12 (*P* > .05). The FBG, C-peptide, and HbA1c levels decreased in all 3 groups during the follow-up period. While the treatment methods did not show any significant differences in terms of the HOMA-IR measured preoperatively and in the postoperative month 12 (*P* = .118 and *P* = .672, respectively), significant differences were, however, found in the 1st, 3rd, and 6th months (*P* = .010, *P* = .001 and *P* = .001). In all the follow-ups, HOMA-IR was found to decrease in comparison with each value measured during the previous follow-up period. According to the post hoc analysis, the reductions in HOMA-IR in the 1st, 3^rd^, and 6th months were greater in the LRYGB and LOAGB groups than in LSG (Table 4).

**Table 4:** Metabolic parameters of groups in each follow-up period.

	LSG	LRYGB	LMGB	*P*
	Mean ± S.D.			
**FBG (mg/dL**)				
Baseline	127.4 ± 49.2	132.8 ± 44.8	143.7 ± 78.4	.089
1st mo	104.2 ± 38.1	104.9 ± 45.3	109.4 ± 34.5	.052
3rd mo	96.1 ± 24.8	95.9 ± 38.1	100.5 ± 28.9	.084
6th mo	94.9 ± 20.2	92.6 ± 17.2	95.3 ± 22.9	.421
1 yr	92.1 ± 22.4	89.5 ± 11.9	91.1 ± 19.8	.589
**C-PEPTID (ng/mL**)				
Baseline	4.9 ± 2.9	5.0 ± 3.2	4.8 ± 2.7	.860
1st mo	4.4 ± 2.9	4.5 ± 3.1	4.3 ± 2.5	.999
3rd mo	4.1 ± 2.9	4.1 ± 2.9	3.8 ± 3.2	.128
6th mo	3.4 ± 2.7	3.5 ± 2.9	3.2 ± 2.9	.497
1 yr	3.1 ± 2.7	3.3 ± 2.7	2.9 ± 2.5	.560
**HbA1C (%**)				
Baseline	6.5 ± 1.5	6.7 ± 1.6	7.1 ± 1.9	.057
1st mo	6.0 ± 1.3	6.1 ± 1.7	6.2 ± 1.0	.051
3rd mo	5.4 ± 0.8	5.5 ± 0.9	5.6 ± 0.9	.094
6th mo	5.5 ± 0.6	5.5 ± 0.8	5.5 ± 0.7	.128
1 yr	5.4 ± 0.6	5.4 ± 0.6	5.5 ± 0.7	.852
**HOMA-IR**				
Baseline	9.3 ± 8.0	10.1 ± 9.0	10.4 ± 7.1	.118
1st mo	5.4 ± 4.0	4.5 ± 2.3	4.4 ± 3.0	**.010**
3rd mo	4.0 ± 2.5	3.1 ± 1.9	2.5 ± 1.6	**.001**
6th mo	2.9 ± 1.6	2.4 ± 1.5	2.2 ± 0.7	**.001**
1 yr	2.3 ± 1.3	2.1 ± 1.0	2.1 ± 0.9	.672
**Hemoglobin (g/dL**)				
Baseline	13.2 ± 1.5	13.6 ± 1.7	13.2 ± 1.6	.151
1st mo	13.3 ± 1.4	13.6 ± 1.3	13.3 ± 1.3	.516
3rd mo	13.1 ± 1.5	13.4 ± 1.2	13.0 ± 1.6	.614
6th mo	13.0 ± 1.5	13.3 ± 1.1	12.9 ± 1.4	.058
1 yr	12.7 ± 1.6	13.0 ± 1.0	12.9 ± 1.5	.591
**TG (mg/dL**)				
Baseline	156.6 ± 69.2	147.4 ± 68.1	175.0 ± 83.3	.089
1st mo	137.1 ± 42.8	130.5 ± 38.9	140.9 ± 43.4	.223
3rd mo	114.4 ± 44.1	104.9 ± 41.9	112.3 ± 44.9	.148
6th mo	109.8 ± 35.8	104.7 ± 32.4	108.8 ± 31.1	.399
1 yr	103.4 ± 40.2	94.5 ± 26.7	104.0 ± 54.7	.383
**LDL (mg/dL**)				
Baseline	160.6 ± 56.9	157.0 ± 52.4	160.3 ± 76.7	.418
1st mo	141.5 ± 56.3	148.1 ± 59.2	144.0 ± 54.1	.825
3rd mo	126.1 ± 38.0	128.1 ± 54.0	127.8 ± 50.1	.097
6th mo	115.1 ± 36.1	113.7 ± 18.1	114.2 ± 25.3	.063
1 yr	105.7 ± 45.2	102.2 ± 22.6	104.4 ± 38.9	.840
**HDL (mg/dL**)				
Baseline	40.3 ± 8.7	40.8 ± 8.4	40.7 ± 9.2	.733
1st mo	41.9 ± 9.9	40.3 ± 8.9	40.5 ± 8.5	.269
3rd mo	47.5 ± 10.6	47.3 ± 10.9	45.5 ± 10.6	.260
6th mo	50.9 ± 10.5	47.1 ± 9.8	45.8 ± 9.0	**.001**
1 yr	55.2 ± 10.4	53.6 ± 10.7	54.5 ± 12.9	.057
**Vitamin B12 (pg/mL**)				
Baseline	446.2 ± 214.3	441.9 ± 178.3	435.7 ± 164.9	.708
1st mo	403.5 ± 106.9	423.3 ± 119.0	426.8 ± 147.3	.072
3rd mo	361.1 ± 185.3	349.2 ± 119.3	408.3 ± 178.0	.117
6th mo	325.3 ± 182.6	288.4 ± 180.8	260.5 ± 101.7	**.001**
1 yr	305.6 ± 139.9	274.2 ± 57.3	274.4 ± 91.3	**.019**
**Folic acid (ng/mL**)				
Baseline	15.1 ± 4.0	15.6 ± 3.2	15.3 ± 3.7	.708
1st mo	13.4 ± 5.3	14.1 ± 4.3	13.9 ± 5.5	.302
3rd mo	12.7 ± 4.1	12.3 ± 4.1	12.6 ± 4.1	.727
6th mo	12.2 ± 3.7	10.4 ± 3.7	10.3 ± 3.4	**.001**
1 yr	12.1 ± 3.5	9.1 ± 3.4	8.8 ± 3.6	**.001**
**Albumin (g/dL**)				
Baseline	4.5 ± 0.2	4.5 ± 0.3	4.5 ± 0.4	.841
1st mo	4.4 ± 0.3	4.5 ± 0.3	4.4 ± 0.4	.536
3rd mo	4.4 ± 0.3	4.5 ± 0.4	4.4 ± 0.4	.758
6th mo	4.4 ± 0.4	4.4 ± 0.4	4.4 ± 0.5	.793
1 yr	4.4 ± 0.3	4.5 ± 0.4	4.5 ± 0.4	.685
**Vitamin D (ng/mL**)				
Baseline	32.9 ± 14.3	33.3 ± 17.9	33.1 ± 14.8	.779
1st mo	33.1 ± 15.4	30.0 ± 15.5	30.3 ± 15.9	.064
3rd mo	30.8 ± 17.9	28.2 ± 18.7	28.6 ± 19.2	.056
6th mo	29.6 ± 19.4	24.3 ± 22.8	23.7 ± 20.1	**.001**
1 yr	28.0 ± 22.7	19.0 ± 17.1	18.4 ± 15.3	**.001**
**PTH (ng/L**)				
Baseline	43.9 ± 27.4	37.6 ± 26.3	38.6 ± 19.6	.206
1st mo	43.8 ± 19.8	40.7 ± 22.8	40.7 ± 25.9	.056
3rd mo	45.2 ± 27.6	42.3 ± 28.1	43.4 ± 29.0	.373
6th mo	49.9 ± 30.5	53.4 ± 20.6	54.9 ± 32.4	.079
1 yr	53.3 ± 24.8	71.7 ± 17.9	68.8 ± 24.5	**.001**
**Iron (µg/dL**)				
Baseline	89.3 ± 32.3	87.9 ± 30.9	88.4 ± 35.0	.706
1st mo	89.6 ± 43.1	84.6 ± 26.7	84.6 ± 28.6	.978
3rd mo	83.2 ± 33.0	75.5 ± 23.1	75.2 ± 30.2	.065
6th mo	79.3 ± 34.4	64.9 ± 17.1	64.5 ± 21.1	**.001**
1 yr	70.4 ± 35.3	49.6 ± 22.8	46.9 ± 23.5	**.001**
**Ferritin (µg/L**)				
Baseline	132.5 ± 54.6	139.2 ± 60.2	136.4 ± 52.7	.771
1st mo	120.8 ± 35.7	123.2 ± 52.1	121.4 ± 45.9	.136
3rd mo	113.3 ± 37.6	113.7 ± 18.1	114.2 ± 25.3	.369
6th mo	101.3 ± 45.6	102.2 ± 22.6	98.9 ± 29.7	.744
1 yr	97.5 ± 44.1	84.4 ± 26.7	81.5 ± 31.8	**.002**

FBG = fasting blood glucose, HDL = high-density lipoprotein, HOMA-IR = the homeostasis model assessment of insulin resistance, LDL = low density lipoprotein, LRYGB = laparoscopic Roux-En-Y gastric bypass, LSG = laparoscopic sleeve gastrectomy, PTH = parathyroid hormone, TG = triglycerides.

Significant differences (*P* < .05).

### 3.5. Lipid profile

There was no significant difference between the surgical methods in the TG or LDL levels measured preoperatively and in the postoperative months 1, 3, 6, and 12 (*P* > .05). The TG and LDL levels were found to constantly decrease in all the follow-up measurements. No significant differences were noted between the surgical methods in the HDL levels measured preoperatively and in the postoperative 1st, 3rd, and 12th months (*P* > .05), with the exception of the postoperative month 6 at which the levels differed significantly (*P* = .001). The mean HDL level in the LSG group was 50.9 ± 10.5, which was higher than that in the other 2 groups (Table 4).

### 3.6. Nutritional profile

Vitamin B12 and folic acid levels maintained their downward trend in all the follow-ups. The surgical methods were found to differ significantly in the vitamin B12 and folic acid levels in the 6th and 12th months (*P* < .05). According to the post hoc analysis, compared with the other 2 groups, the LSG group presented higher mean values of vitamin B12, with a mean level of 325.3 ± 182.6 in the 6th month and 305.6 ± 139.9 in the 12th month. The decline in the LRYGB and LOAGB groups was greater than that in LSG. Compared with the other 2 groups, the LSG group exhibited a higher mean value of folic acid in the 6th and 12th months, with the mean value being 12.2 ± 3.7 and 12.1 ± 3.5 in the 6th and 12th months, respectively. The decline in LRYGB and LOAGB groups was greater than that in LSG (Table 4).

There were significant differences in the vitamin D levels in the 6th and 12th months (*P* = .001 and *P* = .001).The vitamin D levels were found to decrease in each follow-up compared with the previous follow-up period. According to the post hoc analysis, compared with the other 2 groups, the LSG group had higher vitamin D levels in the 6th and 12th months, which were 29.6 ± 19.4 and 28.0 ± 22.7, respectively. The decline in the LRYGB and LOAGB groups was greater than that in LSG. The surgical methods were found to have significant differences in terms of the PTH levels measured in the 12th month (*P* = .001). According to the post hoc analysis, the mean PTH level in the 12th month was 53.3 ± 24.8 in the LSG group, 71.7 ± 17.9 in the LRYGB group, and 68.8 ± 24.5 in the LOAGB group. The rise in the LRYGB and LOAGB groups was greater than that in the LSG group (Table 4).

The surgical methods were also found to differ significantly in terms of the iron levels measured in the 6th and 12th months (*P* < .05). The iron levels in the LSG group in the 6th and 12th months were found to be 79.3 ± 34.4 and 70.4 ± 35.3, respectively, which were higher than those of the other groups. The decline in iron levels in the LRYGB and LOAGB groups was greater than that in LSG. The surgical methods had significant differences in terms of the ferritin level measured in the 12th month (*P* = .002). The decline in the LRYGB and LOAGB groups was greater than that in LSG (Table 4).

### 3.7. Complications

In our study, 10.1% of the cases developed complications. Bile reflux was detected in 7.3% of the patients undergoing LOAGB, 1.8% of the patients undergoing LSG, and 0.9% of those undergoing LRYGB; the rates were found to be significant different (*P* = .006). The groups of surgical methods had significant differences in terms of the incidence rate of Dumping syndrome (*P* = .039), and none of the patients undergoing LSG developed this syndrome. The surgical methods were also found to differ significantly in terms of the presence of internal hernia (*P* = .020). While the groups of patients undergoing LSG and LOAGB did not develop internal hernia, 1.8% of those in the LRYGB group developed the condition. Although there was no statistically significant difference among the groups in terms of staple-line or anastomotic leak, the LSG group (2.4%) had the highest incidence of leak (Table 5).

**Table 5:** Distribution of complications on surgical methods.

	LSG	LRYGB	LMGB	Total	*P*
	N (%)*	N (%)*	N (%)*	N (%)*	
Surgical site infection	3 (0.9)	0 (0.0)	1 (1.0)	4 (0.7)	.591
Bile reflux	6 (1.8)	1 (0.9)	7 (7.3)	14 (2.6)	**.006**
Dumping syndrome	0 (0.0)	2 (1.8)	2 (2.1)	4 (0.7)	**.039**
Internal hernia	0 (0.0)	2 (1.8)	0 (0.0)	2 (0.4)	**.020**
Pulmonary embolism	3 (0.9)	1 (0.9)	1 (1.0)	5 (0.9)	.993
Postoperative bleeding	8 (2.4)	5 (4.6)	3 (3.1)	16 (3.0)	.518
Staple-line or anastomotic leak	8 (2.4)	1 (0.9)	0 (0.0)	9 (1.7)	.208
**Total**	**28 (8.5**)	**12 (11.0**)	**14 (14.6**)	**54 (10.1**)	
	**Mean ± S.D.**				
Operation time (min)	72.3 ± 20.1	123.8 ± 32.3	88.4 ± 6.3		**.001**
Length of hospital stay (d)	5.3 ± 1.3	5.1 ± 1.1	5.0 ± 1.5		.102

LRYGB = laparoscopic Roux-En-Y gastric bypass, LSG = laparoscopic sleeve gastrectomy.

* Column percentage, significant differences (*P* < .05).

Mortality occurred in a total of 4 patients, 3 of whom were operated with the LSG technique and one with the LRYGB technique. Mortality was due to staple-line leak in 3 of the patients and due to pulmonary embolism in the remaining patient. The mean operative time was 72.3 ± 20.1 in LSG, 123.8 ± 32.3 in LRYGB, and 88.4 ± 6.3 min in LOAGB, with the highest operative time being found in the group of patients undergoing LRYGB (*P* = .001). There was no significant difference among the surgical methods in terms of hospital stay (*P* = .102).

## 4. Discussion

In terms of weight loss, the mean EWL% and TWL% values in the 1st and 3rd months were higher in the LOAGB group than in those who underwent LSG and LRYGB, and the mean EWL% and TWL% values in the 6th and 12th months were similar in all 3 groups. In a study conducted in India to evaluate the 6-year-long outcomes in 55 patients undergoing MGB (mini-gastric-bypass) and 68 undergoing LSG, the weight loss in the 1st year was faster in the MGB group and the 2 groups differed significantly in terms of the 1st year weight loss. However, the weight loss in the 6th year was slightly higher in the LSG group, and no significant differences were found between the groups.^[10]^ In a study comparing the 1st year outcomes in France, the EWL% was 71.4 ± 19.0 in the LSG group and 79.3 ± 17.8% in the MGB group at the end of the 1-year period. The TWL% was 34.3 ± 8.4 in the LSG group and 38.2 ± 8.4 in the MGB group. These results were different at statistically significant levels between the groups, and the weight loss in the MGB group was higher at the end of the 1-year period.^[11]^ In our study, by the end of the 1-year period, although there was no statistically significant difference between the groups, the weight loss after 1 year was higher in LOAGB in our study.

In a study of 287 patients in Germany, the EWL% varied between the groups in a statistically significant manner by the end of the 1st year, being 66.2 ± 13.9 in MGB and 57.3 ± 19.0 in LSG.^[12]^ In a study of 160 patients in Italy by Milone et al,^[13]^ the BMI reduction in the first 3–6 months was greater in the MGB group than in LSG.

In a multicentric study, the TWL% in the 6th month was 26.4 in MGB, 24.4 in RYGB, and 25.2 in LSG and there were no significant differences among the groups. Nevertheless, the TWL% values in the 12th month were measured as 32.7, 33.4, and 29.5 in the MGB, RYGB, and LSG groups, respectively, and differed significantly among the groups. In our study, the TWL% values were 30.4 ± 5.7, 27.8 ± 7.2, and 29.1 ± 6.4 in the 6th month and 36.5 ± 6.8, 33.7 ± 9.0, and 35.2 ± 7.7 in the 12th month in the MGB, RYGB, and LSG groups, respectively.^[14]^ In a case study involving 109 patients, Ece et al^[15]^ found that LRYGB achieved greater rates of weight loss in the 1st and 3rd months compared with the LSG technique, whereas no significant differences were found between the 2 techniques in terms of the weight loss in the 6th and 12th months.

In a case study involving 390 patients who were followed up for 3 years to compare the LRYGB and LSG methods, there was no significant difference between the 2 groups in the TWL% and EWL% achieved during the 1st year of follow-up.^[16]^ In another study comparing the LRYGB and LSG methods in 98 patients, the patient data for the 1st and 12th months were compared and no significant differences were found between the 2 techniques in terms of the set of data compared.^[17]^ In a France-based study in which the LRYGB and LSG methods were compared in 86 patients in terms of the 6- and 12-month outcomes, there were significant differences in terms of weight loss.^[18]^

In parallel with the weight loss, HOMA-IR also kept decreasing in each follow-up. The reductions in the 1st, 3rd, and 6th months were greater in the LRYGB and LOAGB groups than in LSG. In the 12th month, similar outcomes were obtained for all of the surgical techniques performed. A study comparing the MGB method with LSG and LRYGB found that the former had a DM remission rate of 93.1% at the end of 1 year, which was higher than that in the other methods.^[14]^ In a study comparing LRYGB and LSG, as in our study, LRYGB showed a faster improvement in the HOMA-IR level in the 1st and 3rd months.^[15]^ In a study of 32 diabetic rats in China, the preoperative HOMA-IR levels were not different between the groups, whereas there were significant differences between the groups in terms of the HOMA-IR measured in the postoperative 8th week. Furthermore, the HOMA-IR levels were found to be lower in RYGB than in the LSG.^[19]^

Regarding the lipid profile, the levels of TG and LDL maintained their downward trend in all follow-ups. However, there was a significant difference among the groups in terms of the mean HDL in the 6th month; the mean HDL was 50.9 ± 10.5 mg/dL in the LSG group, which was higher than that in the other groups. In a study comparing LSG and LOAGB techniques, the serum levels of triglyceride, LDL, and HDL were similar in all follow-ups and the lipid levels were found to have improved significantly.^[10]^ In another study comparing LSG and LOAGB, there was no difference in the preoperative lipid levels, whereas the variance of the 3rd month in the MGB group was greater than that in the LSG group. No significant differences were found in terms of the TG, LDL, and HDL levels measured in the 6th and 12th months.^[13]^ However, in 1 study, the rate of dyslipidemia remission was reported to be consistently lower in the group of patients undergoing LSG than in those undergoing LRYGB and LOAGB.^[14]^ Again, in another study, no significant differences were reported in any of the follow-up periods among the groups in terms of LDL and HDL.^[15]^ A study comparing LSG and LRYGB found a greater reduction in the LDL level at 1-year follow-up in the group of patients undergoing LRYGB than in the LSG group.^[18]^

Vitamin B12 decreased over time in all surgical techniques and was found to be higher in the LSG group in the 6th and 12th months than in the other groups. The decline in the LRYGB and LOAGB groups was greater than that in LSG. Vitamin B12 was found to be significantly lower in the LRYGB group than in LSG, similar to our study in most studies.^[15,17,18]^ Unlike these studies, a study comparing the 3-year outcomes in LSG and LRYGB found no significant difference between the 2 surgical techniques at the end of 1 year.^[16]^

Folic acid levels decreased constantly in all follow-up periods, and significant differences were found among the surgical techniques in the folic acid levels measured in the 6th and 12th months. The decline in the LRYGB and LOAGB groups was greater than that in LSG. Unlike the reported study where it is reported to be, as in our study, 10.6 ± 4.1 in the 6th month and 8.8 ± 3.5 in the 12th month in the LRYGB group and significantly lower than that in the LSG group,^[15]^ there is also another study where the groups did not differ significantly in terms of the values measured in the 1st and 12th months.^[17]^

Vitamin D levels continued to decrease in all follow-up periods. The mean vitamin D levels in the 6th and 12th months were 29.6 ± 19.4 and 28.0 ± 22.7, respectively, in the LSG group, which were higher than those of the other groups. The decline in the LRYGB and LOAGB groups was greater than that in LSG. In another study in which the findings of the postoperative months 1, 3, 6, and 12 were evaluated, as in our study, there was a significant difference only in the 12th month at which the value was 30.5 ± 9.9 ng/mL and higher in the LSG group than in the LRYGB group.^[15]^ Similarly, in a study comparing the findings of the 6- and 12-month follow-ups, the value was found to be 23.3 ± 13.1 in the 6th month and 25.1 ± 9.3 ng/mL in the 12th month and higher in the LSG group than in LRYGB.^[18]^ Unlike these studies, in a study there is no significant difference between the 2 surgical techniques at the end of 1 year.^[16]^ In our study, there was an ongoing upward trend in the PTH levels in all follow-ups and significant differences were found among the groups at the end of the 12-month period. The increase in the LRYGB and LOAGB groups was greater than that in LSG. Studies in France and India found no significant difference.^[16,18]^ Contrary to studies, there is also a study in which the PTH level was found to be higher in LSG with 74.6 ± 18.2 ng/L at 12 months.^[15]^

The LSG group was found to have higher mean levels of iron in the 6th and 12th months than the other groups, with mean iron levels of 79.3 ± 34.4 and 70.4 ± 35.3, respectively. However, the mean level of ferritin in the 12th month was 97.5 ± 44.1 in the LSG group, while it was 84.4 ± 26.7 and 81.5 ± 31.8 in the LRYGB and LOAGB groups, respectively, and there was a significant difference among the groups. The decline in the LRYGB and LOAGB groups was greater than that in LSG. A study of 98 patients in Poland found significant differences among the groups in the mean levels of iron and ferritin in the 1st month, and they were higher in LSG than in the other groups. However, no differences were found among the groups in the 12th month.^[17]^ In a study evaluating the ferritin levels, there were no differences among the groups in terms of their ferritin levels at the 12th month.^[16]^ In another study, the iron level was higher in the LSG group in the 6th month but was higher in the LRYGB group in the 12th month, and the groups differed significantly. In the same study, the LRYGB group was found to have lower levels of ferritin in the 6th month (104.5 ± 98.0 μg/L) and 12th month (97.3 ± 116.6 μg/L), and the groups were found to differ significantly.^[18]^ Similar to our study, in another study too, the LSG group was found to have significantly higher levels of iron at 80.0 ± 15.1 μg/dL in the 6th month and 69.5 ± 16.7 μg/dL in the 12th month.^[15]^

In terms of postoperative complications, LOAGB had the highest incidence rate (7.3%) of bile reflux. None of the patients in the LSG group developed Dumping syndrome. However, while internal hernia formation was not seen in LSG and LOAGB, 1.8% of the patients undergoing LRYGB developed the condition. Although there was no statistically significant difference among the groups in terms of staple-line or anastomotic leak, the highest incidence rate of leaks was seen in the LSG group (2.4%). In a study in which Chiappetta et al^[20]^ compared LRYGB and LOAGB, the incidence rate of Dumping syndrome was higher in LRYGB at 19.0% and the incidence rate of gastroesophageal reflux (GER) was higher in LOAGB at 11.8%. According to a meta-analysis comparing LSG and LOAGB, the latter demonstrated lower rates of leak (*P* = .020) and GER (*P* = .006) than the former.^[21]^ There is also a study indicating a significantly higher rate of Dumping syndrome in LOAGB (10.0%) than in LSG.^[10]^ While a study has shown a higher rate of leak in LSG than in LOAGB,^[12]^ another study has shown that there is no significant difference between the 2 techniques in terms of the leak incidence rate.^[11]^ In a study of 1107 patients that compared the occurrence of complications, the incidence rate of bile reflux was the highest in MGB at 0.4%, whereas the incidence rate of Dumping syndrome was the highest in the LRYGB at 2.7%, followed by 5.9% in LOAGB and 0.0% in LSG. The LRYGB group had the highest rate of internal hernia formation at 2.0%, whereas the highest incidence rate of leak was found in LSG (1.5%).^[22]^

The operative times in our study were 72.3 ± 20.1 (LSG), 123.8 ± 32.3 (LRYGB), and 88.4 ± 6.3 (LOAGB) min, with the highest operative time being found in LRYGB and the lowest in LSG. Unlike the studies showing significantly higher operative times in LRYGB,^[14,22,23]^ which is similar to that reported in our study as well, a German study comparing LOAGB and LSG in patients reported a shorter operative time in LOAGB (81.7 ± 25.3) than in LSG (112.1 ± 33.5).^[12]^

## 5. Study limitations

The strength of the present study is that it is 1 of the few studies in the literature comparing the 3 techniques. Its limitation is that the study reports the outcomes of only a 1-year follow-up period.

## 6. Conclusion

All 3 techniques were found to be successful in facilitating weight loss at the end of the 1st year. LRYGB and LOAGB were superior to LSG in terms of remission from DM during the first 6 months, whereas LSG was superior to the other methods in terms of nutritional deficiencies. Despite being more advantageous in terms of operative time when compared with the LRYGB technique, LSG and LOAGB were disadvantageous because of the higher rates of leakage and mortality in the LSG technique and the higher rate of bile reflux in the LOAGB technique. Further studies with longer follow-up periods are needed, and we are continuing our patient follow-ups for our future studies.

## Author contributions

**Conceptualization:** Haci Murat Cayci, Hasan Cantay, Kenan Binnetoglu, Umut Eren Erdogdu.

**Data curation:** Haci Murat Cayci, Hasan Cantay, Kenan Binnetoglu, Umut Eren Erdogdu, Yurdakul Deniz Firat.

**Formal analysis:** Haci Murat Cayci, Hasan Cantay, Umut Eren Erdogdu.

**Funding acquisition:** Haci Murat Cayci, Hasan Cantay, Umut Eren Erdogdu.

**Investigation:** Haci Murat Cayci, Hasan Cantay, Kenan Binnetoglu, Yurdakul Deniz Firat.

**Methodology:** Hasan Cantay, Kenan Binnetoglu.

**Project administration:** Haci Murat Cayci, Hasan Cantay, Umut Eren Erdogdu.

**Resources:** Hasan Cantay, Kenan Binnetoglu, Yurdakul Deniz Firat.

**Software:** Haci Murat Cayci, Hasan Cantay, Umut Eren Erdogdu.

**Supervision:** Haci Murat Cayci, Hasan Cantay.

**Validation:** Hasan Cantay, Kenan Binnetoglu, Yurdakul Deniz Firat.

**Visualization:** Hasan Cantay, Yurdakul Deniz Firat.

**Writing – original draft:** Hasan Cantay.

**Writing – review & editing:** Haci Murat Cayci, Hasan Cantay, Umut Eren Erdogdu.
